# Eliminating Animal Products from the Diet Puts Dutch Older Adults at Risk of Micronutrient Deficiencies: A Simulation Study

**DOI:** 10.1016/j.cdnut.2026.109446

**Published:** 2026-07-25

**Authors:** Jos Borkent, Pol Grootswagers, Liliana G Mendoza Valbuena, Marian de van der Schueren

**Affiliations:** 1 Department of Nutrition, Dietetics and Lifestyle, HAN University of Applied Sciences, Nijmegen, The Netherlands; 2 Division of Human Nutrition and Health, Wageningen University, Wageningen, The Netherlands

**Keywords:** sustainability, micronutrients, simulation study, protein transition, nutrition adequacy

## Abstract

**Background:**

Transitioning to more sustainable eating habits with less animal-based protein affects not only protein intake but also micronutrient consumption. Given that older adults are already vulnerable to insufficient micronutrient amounts, such dietary changes could further heighten their risk of deficiencies.

**Objectives:**

We simulated how switching toward a more sustainable eating pattern influenced micronutrient intake in older adults.

**Methods:**

Data from the Dutch National Food Consumption Survey 2019–2021 (community-dwelling older adults, *n* = 607, food intake based on 2-d food diary) were used to simulate 2 flexitarian (40% and 80% meat/fish replacement), pescetarian, vegetarian, and vegan diets. and compared with the estimated average requirement (EAR) or adequate intake based on the Dutch dietary reference values. Nutrient intake was deemed low if >10% of participants fell below the EAR or if the median was below the adequate intake.

**Results:**

In the reference diet, actual micronutrient intake among Dutch older adults was generally adequate, except for riboflavin, vitamin B6, folate, and calcium. Vitamin A and selenium were low in females only. In the simulated flexitarian 40% scenario, no additional nutrients were considered low. In the flexitarian 80%, pescetarian, and vegetarian scenarios, niacin in all, and vitamin A and thiamin in males became low. In the vegan scenario, nearly all micronutrient intakes were low.

**Conclusions:**

Reducing or eliminating animal-based protein sources from the diets of older adults elevates their risk of micronutrient deficiencies.

## Introduction

A Western eating pattern with relatively high consumption of animal-based protein places a large burden on our planet [1]. Therefore, shifting toward a more sustainable eating pattern (in terms of carbon dioxide emission, land use, and water impact [2]) with fewer animal-based products is advised by institutes such as the WHO [3]. Such a pattern consists of more fruits, vegetables, pulses, whole grains, and nuts, with moderate amounts of fish, eggs, poultry, and dairy, while limiting red meat and cheese [4,5].

Although various studies on protein transition focused on protein adequacy [6], other nutritional aspects should also be considered. Animal-based products are not only rich sources of protein but also provide essential vitamins and minerals [7]. A deficiency in vitamin B12, e.g., is a well-documented risk when adopting a vegan diet [8]. Additionally, the Dutch Health Council emphasizes the importance of monitoring vitamin A, vitamin B2, vitamin B12, as well as the micronutrients calcium, iron, and iodine when transitioning to a more plant-based eating pattern [9].

Micronutrients play a crucial role in the aging process as they are necessary for wound healing, maintaining bone health, synthesis of cells, and muscle functioning [10]. In general, older adults already have insufficient intakes of several micronutrients, such as thiamin, riboflavin, calcium, selenium, and magnesium, largely because of low overall food intake [11]. Special attention should thus be paid to guaranteeing sufficient micronutrient intake when adopting a more sustainable diet to support these essential processes.

A few studies have modeled the impact of transitioning to a more sustainable diet on micronutrient intake [12]. These studies were mainly based on replacing one specific food group (for instance, red meat) or focused on increasing certain products, such as legumes, instead of simulating the most common more sustainable eating patterns, such as flexitarian, vegetarian, pescetarian, or vegan. Even more important, most simulation studies were performed in younger adults or did not stratify their results by age group [12]. Therefore, it is unlikely that these findings can be extrapolated to older adults, who are already at risk of inadequate micronutrient intake from their habitual diets [11].

To evaluate the potential risk of micronutrient deficiencies in older adults when transitioning to a more sustainable eating pattern, we conducted a simulation study. We examined the consequences of shifting to flexitarian, pescetarian, vegetarian, and vegan diets on their micronutrient intake amounts.

## Methods

Simulations were made on data of the Dutch National Food Consumption Survey (DNFCS) from the period 2019–2021 [13]. The DNFCS is a cross-sectional periodical survey to assess food intake of the general Dutch population and is conducted by the National Institute for Public Health and The Environment. The database is nationally representative based on age, sex, region, education level, and urbanization level. Besides data on food consumption, self-reported data on sex, age, weight, height, level of education and employment, and lifestyle characteristics were collected.

The DNFCS 2019–2021 included people living at home in the age category 1 to 79 y (*n* = 3570) but for this study, we only used data of those aged 65 y or older (*n* = 607).

The study was conducted according to the guidelines of the Helsinki Declaration. The Medical Ethical Committee of the University Medical Centre Utrecht determined that the study did not require full review under the Medical Research Involving Human Subjects Act, as all survey measurements were noninvasive (reference no. 19-145/C). All participants signed an informed consent form at the first home visit.

### Dietary intake and nutrition composition

Food intake of all participants in the DNFCS was measured by using 2 nonconsecutive 24-h dietary recalls per participant, making use of GloboDiet [14]. Both recalls were performed with a gap of 2 to 6 wk between them, allowing for a more accurate representation of typical dietary intake. Participants aged >70 y filled in a food diary as a memory aid for the 24-h recall 1 d before the interview. Interviews were conducted throughout the year and both during week and weekend days.

Participants aged 65 to 70 y were interviewed twice by telephone. Those aged 70 y or older received 1 in-home visit and 1 phone interview. However, because of the COVID-19 pandemic, some participants were interviewed twice by telephone instead.

Food consumption was noted on product level and categorized by food consumption occasion (3 meals and 4 in-between moments) category by the participants. The Dutch food composition database (NEVO) was used to calculate intake of micronutrients [15].

### Simulations

For the simulations, we used pre-established scenarios [16], including 2 flexitarian scenarios (with either 40% or 80% of meat/fish replaced), a pescetarian scenario (where all meat was replaced but fish remained), a vegetarian scenario (where both meat and fish were replaced), and a vegan scenario (where all animal-based products were replaced). We followed a 2-step approach: we first assessed whether a product is compatible with a scenario; if not, we replaced it randomly by an appropriate alternative.

#### Assessing compatibility and alternatives

For the scenarios, we evaluated ∼1600 unique food codes used in the DNFCS to determine their compatibility with each scenario. This assessment was conducted by a dietitian (JB). Assessment of compatibility for each product was added to the ∼40,000 food items consumed in the DNFCS. Foods that did not fit into a scenario (for instance, a hamburger in a vegetarian scenario) were marked as “to be replaced” and categorized into one of the following food groups: meat or fish, bread with meat, savory snacks, sandwich fillings based on meat/fish/egg, soup, minced meat, yogurt/cream/sweet pudding/dessert/ice cream, dairy-based drinks, sweet snacks, or creams. For each food group, ≤12 alternative products were selected by a dietitian (JB and LGMV) based on their similarity to the items being replaced. In cases where fewer suitable alternatives were available, such as soups and snacks, a smaller number of replacements were used (see TABLE 1, TABLE 2).

**TABLE 1:** Alternatives used for replacements shown per food group, for vegetarian scenarios (flexitarian 40%/80%, pescetarian, and vegetarian)

Food group	Replacements
Meat or fish^1^	Chicken egg
	Mozzarella cheese
	Vegetarian vegetable balls/burgers based on soy^2^
	Vegetarian schnitzel, enriched with iron and vitamin B12
	Vegetarian vegetable balls/burgers based on soy, enriched with iron and vitamin B12
	Vegetarian vegetable balls/burgers based on soy^2^
	Vegetarian burger with cheese
	Vegetarian vegetable balls/burgers based on soy^2^
	Vegetarian schnitzel (based on milk) fortified with iron
	Gouda cheese 48+
	Sausage roast—vegetarian based on peas^2^
	Vegetarian meatballs/burgers based on soy/wheat, enriched with iron and vitamin B12
Sandwich fillings based on meat or fish	Egg salad
	Gouda cheese 48+
	Peanut butter
	Spread sweet average
	Hummus
	Sausage sandwich—vegetarian enriched with iron and vitamin B12
	Cheese salad
	Cream cheese
	Chicken egg
	Cheese 30+
	Cheese spread
	Chocolate sprinkles
Bread with meat	Cheese pastry
	Baguette cheese-onion
	Bread currant
	Roll white soft
Minced meat	Lentils green/brown boiled
	Quorn minced meat^2^
	Vegetarian minced meat enriched with iron and vitamin B12
	Quorn pieces^2^
	Minced meat, vegetarian, based on soy^2^
	Vegetarian meatballs/burgers based on soy/wheat, enriched with iron and vitamin B12
Soup	Soup clear with vegetables and noodles
	Soup clear vegetables
	Soup thickened vegetables
	Soup thickened no filling
Savory snack	Crisp potato
	Cocktail snacks nibb-it
	Cassave crackers
	Falafel

See Appendix 1 for micronutrient content.

1 Fish is allowed in pescetarian scenario.

2 Nonfortified option.

**TABLE 2:** Alternatives used for replacements shown per food group, for vegan scenario

Food group	Replacements
Meat, fish, meat replacements based on dairy/egg, eggs (dinner), cheese (dinner)	Lentils green/brown boiled
	Tofu
	Falafel
	Vegetarian meatballs/burgers based on soy/wheat, enriched with iron and vitamin B12
	Sausage roast—vegetarian based on peas^1^
	Vegetarian meatballs/burgers based on soy/wheat, enriched with iron and vitamin B12
	Vegetarian meatballs/burgers based on soy/wheat, enriched with iron and vitamin B12
	Strips/pieces vegetarian based on soy/wheat enriched with iron and vitamin B12
	Pieces/chunks vegetarian based on soya/wheat^1^
	Vegetable balls/burgers vegetarian based on soya^1^
	Vegetarian meatballs/burgers based on soy/wheat, enriched with iron and vitamin B12
	Vegetarian schnitzel based on soy/wheat, enriched with iron and vitamin B12
Sandwich fillings based on meat, fish, cheese, eggs (breakfast and lunch)	Peanut butter
	Sausage luncheon meat vegetarian^1^
	Hummus naturel
	Sesame paste tahin
	Sausage luncheon—vegetarian enriched with iron and vitamin B12
	Hummus natural
	Bacon pieces vegetarian unprepared^1^
	Bacon pieces vegetarian unprepared^1^
	Plant-based alternative to Gouda cheese based on coconut oil^1^
	Spread sweet averaged
	Peanut butter
	Sausage luncheon meat vegetarian^1^
Bread with meat	Cheese pastry
	Baguette cheese-onion
	Bread currant
	Roll white soft
Minced meat	Lentils green/brown boiled
	Quorn minced meat^1^
	Vegetarian minced meat enriched with iron and vitamin B12
	Quorn pieces^1^
	Minced meat, vegetarian, based on soy^1^
	Vegetarian meatballs/burgers based on soy/wheat, enriched with iron and vitamin B12
Soup	Soup clear with vegetables and noodles
	Soup clear vegetables
	Soup thickened vegetables
	Soup thickened no filling
Savory snack	Crisp potato
	Cocktail snacks nibb-it
	Cassave crackers
	Falafel
Drink with dairy	Tea
	Coffee
	Drink soya original Alpro fortified with calcium and vitamins
	Water
Milk, yogurt, cream sweet, custard, pudding, desserts, ice cream	Drink soy with sugar, fortified with calcium and vitamins
	Drink almond unsweeted^1^
	Rice drink with calcium and vitamins
	Drink soy with sugar, fortified with calcium and vitamins
	Drink soya chocolate with sugar, fortified calcium and vitamins
	Drink oat without sugar fortified with calcium and vitamins
Cream	Cream based on veg oil Alpro cuisine
	Cream based on veg oil Alpro Cuisine Light
Sweet snacks	Turkish delight
	Popcorn sweet, popped without oil
	Chocolate dark w hazelnuts
	Wafer galette

See Appendix 1 for micronutrient content.

1 Nonfortified option.

#### Randomly replacing

Food items that did not fit to an eating pattern (marked “to be replaced,” like fish in a vegetarian scenario) were randomly replaced by one of the alternatives within the same food group in a gram-for-gram way. This was done by changing the original food code in the DNFCS for the new food code that corresponds to the products in TABLE 1, TABLE 2. For instance, a hamburger (food code 1435) does not fit into any eating pattern and could be, depending on a random number, replaced in a vegetarian scenario by the food code of a chicken egg (83). Food codes of items that fitted the eating pattern were left unchanged.

To simulate the models, all separate consumed items in the DNFCS (39,923) were assigned a random number between 0 and 1. This randomization was used to enable the use of multiple alternatives rather than a single fixed option, which would be unrealistic. Replacement options within a category were equally assigned. For instance, in the category “meat or fish,” 12 alternatives were used. The first replacement option (chicken egg) was allocated to the random numbers 0 to 0.083, the second option (Mozzarella cheese) to the random number 0.083 to 0.167, etc. In case of fewer options (2, 3, 4, or 6), all options were equally assigned.

In case of the flexitarian scenario, random number 0 to 0.6 (flexitarian 40%) and 0 to 0.2 (flexitarian 80%) were not replaced. The remaining random numbers (0.6–1.0 for flexitarian 40% and 0.2–1.0 for flexitarian 80%) were equally assigned to the replacement options as described above. R-syntax for replacement is added in Appendix 2.

To make our simulations as realistic as possible, we adhered closely to typical Dutch dietary patterns. Therefore, in the flexitarian, pescetarian, and vegetarian scenarios, we replaced meat and fish (except in the pescetarian scenario) primarily with cheese and processed meat alternatives and only to a small extent with legumes [16,17].

For consumers, both fortified and unfortified processed meat and dairy alternatives are available in supermarkets. To create a realistic balance between these product types in our scenarios, we examined the micronutrient content of 98 meat and dairy alternatives sold in the 2 largest supermarket chains in the Netherlands, Jumbo and Albert Heijn. More than half of the meat replacements for warm meals and nearly all plant-based drinks were fortified (mostly by adding vitamin B12 and vitamin D), whereas meat/cheese replacements on bread were rarely fortified. On the basis of the supermarket inventory, we applied NEVO codes for fortified options in our simulation for more than half of the meat replacements at hot meals (60%) and for nearly all dairy replacements (85%), but only for a small proportion of vegan sandwich fillings (20%). Certain options (for instance, vegetarian vegetable balls/burgers based on soy) were used more often within one category to obtain the right proportion of fortified/nonfortified options (see TABLE 1, TABLE 2). Micronutrient content of all replacements can be seen in Appendix 1.

### Reference values

The Dutch guidelines for micronutrients were used to determine which portion of the population had inadequate micronutrient intakes [18]. We applied the sex-specific estimated average requirements (EARs) for vitamins A, B1, B2, niacin, B6, folate, and B12, as well as the minerals copper, iron, and zinc. Because no EARs are available for vitamin E and the minerals calcium, iodine, magnesium, phosphorus, potassium, and selenium, we used adequate intakes instead (see Table 3). Vitamin D intake was reported but not assessed against the EAR as reference values are based on intake with supplementation [18]. Vitamin C was not incorporated in the analyses as it is almost completely derived from products that do not contain protein. Iodine assessment was omitted because of the limited availability of data on iodine content in the NEVO table.

**TABLE 3:** Daily requirements of micronutrients for older adults (aged ≥65 y) based on Dutch dietary reference values (2018)

Estimated average requirements (EAR)	Adequate intake
Vitamin A (RAE): ♂: 615 μg; ♀: 525 μg	Vitamin E: ♂: 13 mg; ♀: 11 mg
Thiamin: 0.072 mg/MJ	Calcium ♂: 1200 mg^1^
Riboflavin: 1.3 mg	♀ <70 y: 1100 mg ♀ ≥70 y: 1200 mg
Niacin: 1.3 mg/MJ	Magnesium: ♂: 350 mg; ♀: 300 mg
Vit B6: ♂: 1.3 mg; ♀: 1.1 mg	Phosphorus: 550 mg
Folate: 200 μg	Potassium: 3.5 g
Vit B12: 2.0 μg	Selenium: 70 μg
Vit C: ♂: 60 mg; ♀: 50 mg	
Copper: 0.7 mg	
Iron: 6 mg	
Zinc: ♂: 6.4 mg; ♀: 5.7 mg	

RAE = Retinol Activity Equivalents.

1 Only for age ≥70 y.

Micronutrient intake was evaluated based on the method outlined in the DNFCS [13]. A micronutrient was considered to have a low intake on a population level if >10% of the population did not meet the EAR. However, for nutrients assessed using adequate intake, such conclusions cannot be drawn. Instead, it is generally assumed that intake is sufficient when the median intake (P50) exceeds the adequate intake. When P50 is below the adequate intake, no statement can be made on that micronutrient [19].

### Data analyses

All analyses were performed with R (R foundation) (4.2.3). Characteristics of included participants of the DNFCS were described by using means with SDs for normally distributed data, medians with 25th to 75th percentiles for nonnormally distributed data, and counts with percentages for categorical variables. Normality was checked by using qq-plots and stem-and-leaf diagrams.

To compare micronutrient intake with the sex-specific reference values, we used the R package Statistical Program to Assess Habitual Dietary Exposure (SPADE) version 4.1 [20]. SPADE is a package developed by the Dutch National Institute for Public Health and The Environment, especially to analyze the DNFCS. SPADE estimates habitual intake based on repeated short-term dietary assessments, such as the two 24-h recalls used in the DNFCS. It also provides opportunities to stratify data on sex and age and to add weighting factors to make data representative. SPADE provides a distribution of habitual intake on population level expressed in percentiles (for instance, p10, p50, and p90).

A 1-part model was applied for all nutrients, as dietary supplements were not included. We chose not to include supplements because our aim was to show changes in dietary intake and not to focus on total vitamin adequacy within the population. All data were analyzed stratified by sex. Weighting factors (sociodemographic factors, week/weekend days, season) were used to make data nationally and seasonally representative.

Micronutrient intake was visualized using boxplots, where the box represents the 25th, 50th, and 75th percentiles (p25, p50, and p75), and the whiskers indicate the 10th and 90th percentiles (p10 and p90). Changes of micronutrients for each scenario compared with the original diet were shown by using dot plots.

## Results

Of the 607 included participants, 51% were males, median age was 71 in males and 69 in females, and mean BMI was almost 27 kg/m^2^. Of males, 41% had a high educational level; in females this was 27% (Table 4).

**TABLE 4:** Characteristics of participants (aged ≥65 y) included from the Dutch National Food Consumption Survey

Characteristic	Male	Female
	*n* = 311 (51%)	*n* = 296 (49%)
Age (y)	71 (68–84)	69 (67–73)
BMI (kg/m^2^)	26.9 (±4.5)	26.8 (±5.4)
Education level^1^		
Low	96 (31%)	132 (45%)
Middle	89 (29%)	83 (28%)
High	126 (41%)	81 (27%)
Number of food replacements		
Flexitarian 40%	771	596
Flexitarian 80%	1405	1010
Pescetarian	1355	901
Vegetarian	1709	1227
Vegan	4754	4058
Percentage plant-based protein of total protein		
Reference diet	39.0% (30.7–46.8)	37.7% (28.0–46.4)
Flex 40	43.7% (33.9–55.4)	42.6% (32.6–54.0)
Flex 80	52.2% (40.5–65.3)	49.7% (39.2–62.5)
Pescetarian	54.3% (42.1–67.0)	51.9% (39.2–63.8)
Vegetarian	59.1% (45.2–70.0)	54.2% (42.9–65.0)
Vegan	99.3% (98.3–99.8)	99.3% (98.3–99.8)
Kcal intake		
Reference diet	2197 (1955–2463)	1751 (1545–1969)
Flexitarian 40%	2213 (1979–2472)	1760 (1552–1979)
Flexitarian 80%	2211 (1970–2474)	1761 (1550–1984)
Pescetarian	2211 (1971–2499)	1761 (1547–1987)
Vegetarian	2233 (1984–2509)	1770 (1555–1998)
Vegan	2180 (1937–2450)	1747 (1540–1964)

Data are shown as median (25th–75th percentile), mean (with SD), or as number/percentage. Data (except kcal intake) are based on unweighted data, so no adjustments were done to make this table national representative.

1 Education level was categorized into low (primary education, lower vocational education, advanced elementary education), middle (intermediate vocational education, higher secondary education), and high (higher vocational education and university).

Of the total of 39,923 consumed items, the least items (*n* = 1367) were replaced in the flexitarian 40% scenario, and the most (*n* = 8812) in the vegan scenario. All scenarios included less animal-based protein than the reference scenario, but the goal of 60% plant-based proteins as advised by the Dutch Health Council [9] was only reached in the vegetarian diet (males ∼60%, females ∼55% plant-based protein) and the vegan diet (close to 100% plant-based protein, not fully as some low-protein foods such as cake were not replaced). Daily energy intake increased slightly in the flexitarian, pescetarian, and vegetarian scenario (∼100 kcal) but was stable in the vegan scenario (see Table 4).

### Micronutrient intake

**FIGURE 1:**
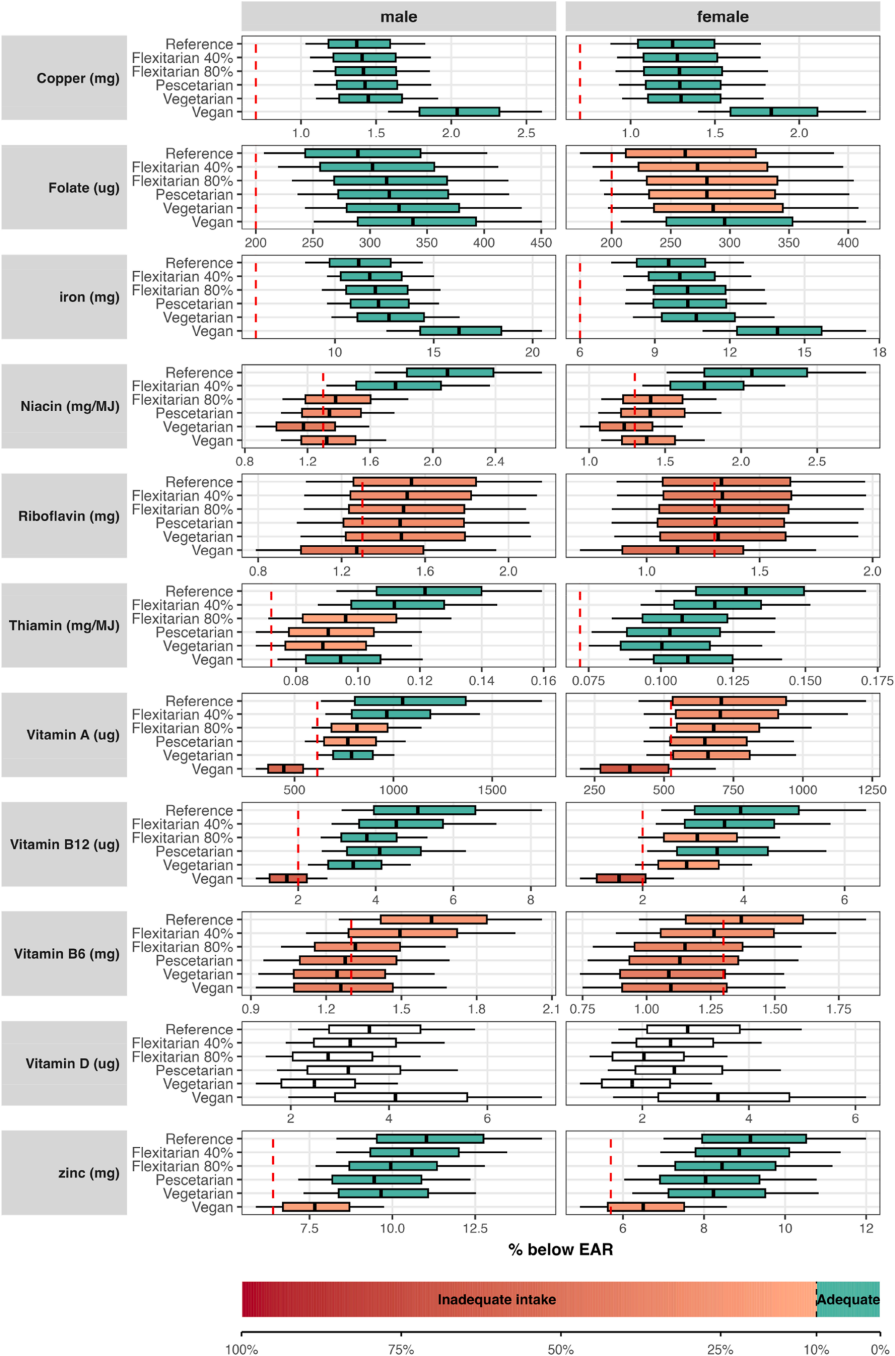
Vitamin intake with corresponding EAR cut-off values, stratified by scenario and sex. Vitamin D is not assessed against EAR as this number is based on intake with supplements. EAR, estimated average requirement.

**FIGURE 2:**
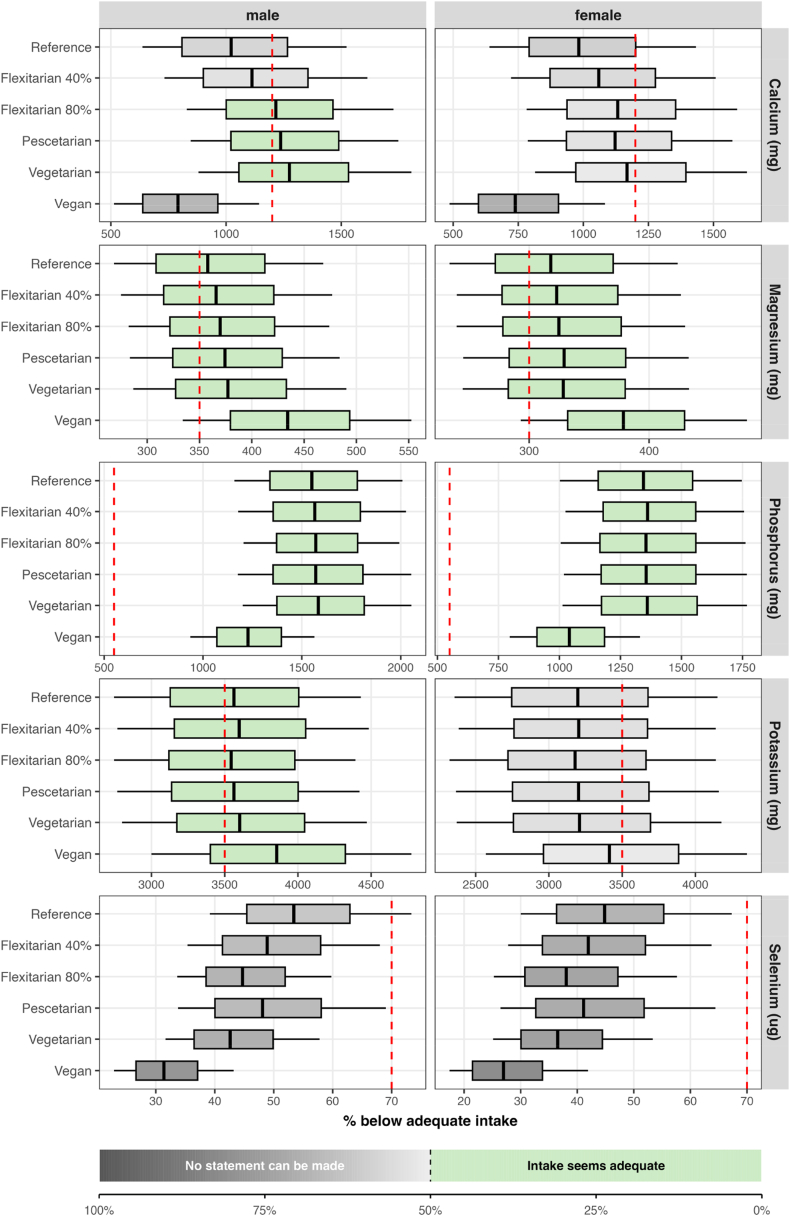
Micronutrient intake with corresponding adequate intake cut-off values, stratified by scenario and sex.

In the reference diet, most micronutrients were consumed sufficiently, except riboflavin, vitamin B6, folate (both sexes), and vitamin A in females (Figure 1). Based on adequate intake, most micronutrients were sufficiently consumed, but no statements could be made for calcium and selenium (both sexes) and potassium in females (Figure 2).

**FIGURE 3:**
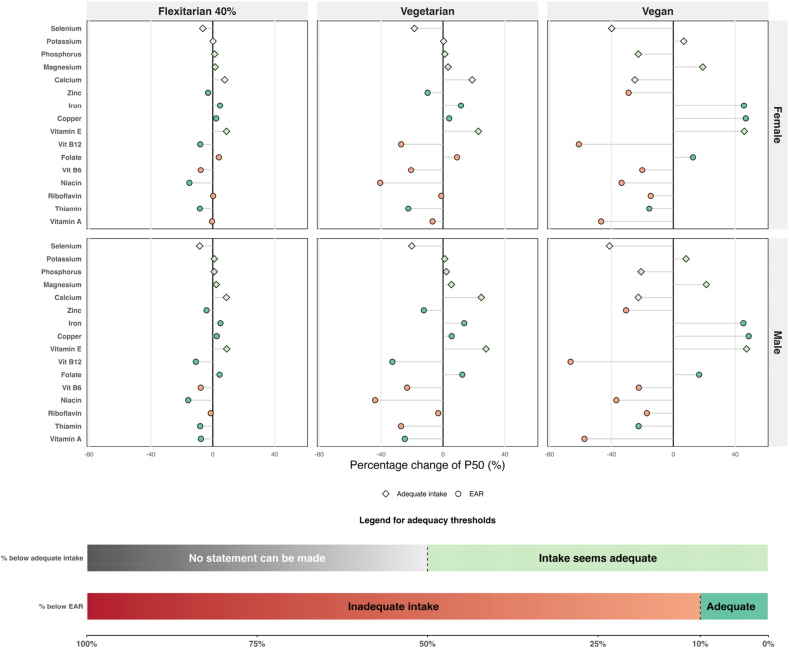
Changes in micronutrient intakes (p50) per simulated diet (flexitarian 40%, vegetarian, and vegan) compared with habitual diet in older males and females.

In the flexitarian 40% scenario, intake of most micronutrients decreased, but no additional micronutrients were too low. In the scenarios with higher plant-based proportions (flexitarian 80%, pescetarian, and vegetarian), the intake of nearly all micronutrients decreased. In addition to the previously mentioned micronutrients, the intake of vitamin A (males), thiamin (males), and niacin (both sexes) became too low. Vitamin B6, which was already low in the reference diet, was now low in >50% of all older adults. In contrast, calcium intake (based on adequate intake) became sufficient in males (FIGURE 2, FIGURE 3).

In the vegan scenario, nearly all micronutrients were low. A sufficient intake was only seen for thiamin, folate (which was low in the reference diet), copper, and iron (all based on EAR) and vitamin E, magnesium, phosphorus (both sexes), and potassium (only in males).

## Discussion

This simulation study among older adults demonstrates that a modest reduction in meat and fish is feasible without introducing extra risks of low micronutrient intake. However, removing substantial amounts of meat and fish leads to a decrease in the intake of key nutrients, particularly vitamin A and B vitamins and zinc. A simulated fully vegan scenario showed a risk of inadequate consumption of nearly all analyzed micronutrients.

Our results align closely with a previous simulation study on the DNFCS, which modeled a “no meat/no dairy” diet in adults aged 18 to 69 y (30% replacement, comparable with our flexitarian scenario and 100% replacement, comparable with our vegan scenario) [21]. Similar declines to those observed in our study were noted for zinc, vitamin A, thiamin, and vitamin B12, whereas concentrations of iron and vitamin D increased (no other micronutrients were assessed). In the 30% replacement scenario, these lower intakes did not result in an additional proportion of participants at nutritional risk (except for vitamin A). In their 100% replacement scenario, large groups did become at risk of low intakes. In line with their 30% replacement scenario, our data suggest that substituting a small portion of animal-based products in the diet is feasible without a strong decline in micronutrient intake.

A recent systematic review indicated that only one trial has examined micronutrient intake when transitioning to more sustainable eating patterns [12]. This trial (*n* = 136; age 20–69 y) assessed micronutrient intake—including vitamin B12, iron, zinc, folate, and iodine—when participants shifted from a 70/30 animal-to-plant protein ratio to 50/50 and 30/70 ratios, primarily by using unprocessed alternatives [22]. Comparable with our results, vitamin B12 significantly decreased by 30% and 55%, zinc declined by approximately 10% to 15%, whereas folate and iron intakes increased in their more plant-based diets.

Observational studies that investigated micronutrient intake among vegetarian and vegan populations were not always in line with our simulated observations. Consistent with our study, these studies show that vegetarian and vegan diets generally provide higher concentrations of folate, similar concentrations of iron, and lower concentrations of vitamins A and B12 compared with omnivorous diets [12]. However, unlike our simulation—which observed significant reductions in several B vitamins—observational studies report similar or even higher intakes of thiamin, riboflavin, niacin, and vitamin B6 in vegetarian and vegan diets [[23], [24], [25]]. These B vitamins are crucial for energy metabolism [26] and for the physical functioning of older adults [27,28]. This discrepancy in vitamin B intake between actual dietary data and our simulation highlights an important limitation of our approach: animal-based products were substituted directly with their plant-based counterparts (often meat analogs), which overlooks the broader differences in dietary habits typically observed between these groups—such as substantially higher intakes of legumes, whole grains, and vegetables [29].

Calcium intake in the reference scenario was low. Shifting dietary patterns toward greater dairy consumption in place of meat—e.g., replacing ham with cheese on sandwiches—reduced the prevalence of inadequate calcium intake. This is particularly relevant for older adults, as calcium is essential for maintaining bone health and preventing osteoporosis, as well as supporting hormonal regulation and vascular function [30]. The relatively high calcium intake observed in our simulations may be explained by the inclusion of cheese products among the substitutes. However, it remains uncertain whether such a shift would occur in everyday practice. Previous studies have reported mixed findings regarding whether individuals following vegetarian diets consume more dairy or cheese than omnivorous diets [31,32].

Iron intake remained high and even increased, with the highest intake observed in the vegan group. That observation replicates those of previous simulation studies [21,33] and of other cohorts that assessed vegan eating patterns [[23], [24], [25]]. In all our scenarios, we included fortified meat replacers as substitutes for meats eaten in a warm meal context. In the Netherlands, fortified meat replacers often contain more iron (±3 to 5 mg/100 g) than their meat counterparts. However, fortification likely varies across countries and products, as there are no European Union regulations on (mandatory) fortification. In the vegan scenario, soy drinks were often introduced as a substitute for dairy drinks. Soy drinks naturally contain ∼1 mg iron per serving of 250 mL [34], whereas dairy drinks do not contain iron, leading to additional increases in the estimated iron intake on a vegan diet. Importantly, iron from plant-based sources, including the fortified meat, consists exclusively of nonheme iron [15], which has a poorer bioavailability [35]. Research showed wide ranges for bioavailability with superior availability of heme iron (15%–35%) [36] compared with nonheme iron (0.7%–22.9%) [37]. Bioavailability of nonheme iron is strongly dependent on an individual iron status [38], which was not available in our data. Therefore, despite the higher intake, it remains unclear whether sufficient iron would be available in more sustainable eating patterns.

Albeit often overlooked, zinc deficiency is one of the most common deficiencies in those with a vegan eating pattern [25]. Our observed decreased intake of zinc while iron remained high might seem remarkable as zinc and iron content in products are correlated with each other, even in plant-based products [39]. However, because processed vegan alternatives are often fortified with iron but not with zinc, this discrepancy is plausible. In the current Western eating pattern, zinc is mainly found in animal-based sources [40]. Plant-based sources of zinc are legumes, such as lentils [15]. For older adults, adequate zinc intake is of importance as it is needed for wound healing, immune function, maintaining bone health, muscle protein synthesis, and taste acuity [41], and zinc fortification or active legume promotion in vegan diets might be considered.

Vitamin B12 intake was lower in each progressively more plant-based scenario, but only fell below thresholds in the vegan scenario. Cohort studies have shown that not only vegan but also those who are following a vegetarian eating pattern are at risk [42]. Plant-based protein alternatives such as nuts, legumes, and soy do not contain any natural source of vitamin B12 [15]. It is therefore commonly advised to use a vitamin B12 supplement when eating vegan [43]. Although meat replacements for warm meals are mostly B12-fortified in the Netherlands, cold-cut replacements are not. In general, the micronutrient profile of meat replacements is not comparable with the pattern of products they aim to replace; meat naturally provides various micronutrients, whereas their replacements are mostly fortified with only iron and vitamin B12. These discrepancies might be considered by food industries and regulatory organizations to better align the nutrient profiles of vegan substitutes with the animal products they replace.

A strength of our modeling approach is the large number of substitutes that we randomly selected from at each replacement, minimizing the risk of single products exerting pronounced effects on micronutrient intakes. In our modeling, we deliberately stayed close to the current eating pattern, as we expect that to be a more realistic population change than diet-wide changes in meal choices. However, this assumption introduces risk to the external validity of our work. In reality, eating a more plant-based diet could be much different than the traditional diet in terms of eating behavior. For instance, it is possible that consuming less animal-based protein will be achieved by eating more mixed meals instead of traditional Dutch meals consisting of a carbohydrate source, vegetables, and a piece of meat or fish, with consequential effects on micronutrient intake. For instance, our observation of dramatically reduced intakes of vitamin A and most B vitamins could be mitigated by an increased consumption of vegetables, legumes, and nuts in a vegan pattern. That idea is supported by cohort studies, showing less pronounced lower micronutrient intakes in vegetarian and vegan diets [24,25]. Thus, our simulation should be interpreted primarily as a reflection of how micronutrient intake might decline when meat and dairy are replaced by processed alternatives, without changes to overall eating patterns.

Another limitation of our study is that dietary intake was self-reported. Although the DNFCS methodology includes multiple quality checks, underreporting or overreporting of intake may still introduce some bias. This is especially relevant for older adults, where memory difficulties can reduce the accuracy of 24-h recall data. On the basis of a previous analysis of this dataset, underreporting in the age group ≥65 y was estimated at 5% in males and 13% in females, which is lower than the 17% estimated for the general DNFCS population [13]. In addition, we used the same underlying data in all our scenarios, so this bias is equal in all scenarios. However, some micronutrient intakes that appear too low might, in reality, be sufficient when accounting for underreporting.

In this study, we showed how micronutrient intake differed for each scenario. Although large differences were sometimes observed between scenarios, no *P* value testing or differences with 95% confidence intervals were calculated. This was not possible because, after the application of SPADE, only the modeled usual intake distributions for each scenario were available, whereas the individual-level paired data linking observations across scenarios were no longer retained. As a result, standard inferential procedures, such as hypothesis testing and the calculation of *P* values, could not be performed.

Finally, simulation studies are inherently theoretical, and the feasibility of adopting a different dietary pattern may be limited, particularly among older adults. However, the younger generations that are open to eating flexitarian, pescetarian/vegetarian, or even vegan will one day become the group of older adults. In addition, the WHO does not restrict recommendations to reduce animal-based protein consumption to younger or middle-aged populations [3]. Our findings suggest that if older adults do reduce their animal-based protein intake, careful monitoring of micronutrient consumption is essential, as current intake amounts are already low and may decline further.

In our study, we did not include supplements, as our aim was to examine how micronutrient intake from foods changed across different scenarios. The proportion of the population at risk of inadequate micronutrient intake may therefore be lower in reality, as a large share of the population currently uses vitamin supplements [44,45] and this is even more pronounced in those consuming a vegan eating pattern [46]. In our data, 60% used a supplement (exact content not indicated), so it is likely that inadequate micronutrient intake after including supplements would be lower.

In conclusion, our simulated scenarios suggest that a shift toward more plant-based diets may increase the prevalence of inadequate micronutrient intakes in older adults. Intakes of the nutrients vitamin A, B vitamins, calcium, selenium, and zinc are most at risk when older adults shift to more plant-based dietary patterns. Future research should confirm these findings in real-life settings.

## Author contributions

The authors’ responsibilities were as follows – JB, PG, MdvdS, LGMV: designed research; JB, LGMV: analyzed data or performed statistical analysis; JB, LGMV, PG, MdvdS: wrote the paper; MdvdS: had primary responsibility for final content; all authors: read and approved the final manuscript.

## Data availability

Data described in the manuscript, code book, and analytic code will be made available upon request. We invite other researchers to contact the corresponding author to collaborate on performing simulations on the DNFCS and to work with our latest syntaxes on this topic.

## Declaration of Generative AI and AI-assisted technologies in the writing process

During the preparation of this work, the authors used ChatGPT to improve grammar. After using this tool/service, the authors reviewed and edited the content as needed and take full responsibility for the content of the publication.

## Funding

This research was partly funded by the Taskforce for Applied Research (grant number: RAAK.MKB16.012), part of the Netherlands Organization for Scientific Research (NOW), financed by the Dutch Ministry of Education, Culture and Science and by a fund of the Dutch Dairy Association. The funders had no role in the design, analyses, and writing or the decision to submit the manuscript.

## Conflict of interest

The author reports no conflicts of interest.
