# Supplementary material for: Eliminating Animal Products from the Diet Puts Dutch Older Adults at Risk of Micronutrient Deficiencies: A Simulation Study
Source: Curr Dev Nutr. 2026 Jul 25;10(8):109446. doi: 10.1016/j.cdnut.2026.109446 (PMC13487607; doi:10.1016/j.cdnut.2026.109446)
Supplement: Supplementary file 1 [file mmc1.docx]

**Title**: Appendix 1: Name (with NEVO codes) of products used as alternatives with corresponding micronutrient content.

Appendix of the study “Eliminating animal products from the diet puts Dutch older adults at risk of micronutrient deficiencies: A simulation study”

Authors: Borkent, Grootswagers, Mendoza Valbuena, de van der Schueren.

| **Name and nevo code** | **Vit A (Ug)** | **Vit D (Ug)** | **Vit E**  **(mg)** | **Thiamin (Mg/mj)** | **Riboflavin (Mg)** | **Vit B6 (Mg)** | **B12 (Ug)** | **Niacin (Mg/mj)** | **Folate (Ug)** | **Vit C (mg)** |
| --- | --- | --- | --- | --- | --- | --- | --- | --- | --- | --- |
| Chicken egg (83) | 194 | 1,1 | 3,1 | 0,07 | 0,38 | 0,089 | 1,49 | 3,1 | 81,5 | 0 |
| Mozzarella cheese  (1955) | 271 | 0,2 | 0,4 | 0,03 | 0,27 | 0,1 | 1,7 | 0,4 | 20 | 0 |
| Vegetarian vegetable balls/burgers based on soy, enriched with iron and vitamin B12  (5551) | 0 | 0 | 4,4 | 0,05 | 0,09 | 0,128 | 0,66 | 0,4 | 41,5 | 0 |
| Vegetarian schnitzel, enriched with iron and vitamin B12 (5565) | 1 | 0 | 3,1 | 0,03 | 0,1 | 0,013 | 0,13 | 0,2 | 9,1 | 0 |
| Vegetarian vegetable balls/burgers based on soy* (5549) | 8 | 0 | 2,3 | 0,08 | 0,08 | 0,121 | 0 | 0,5 | 48,1 | 3 |
| Vegetarian burger with cheese (5566) | 68 | 0,1 | 2,3 | 0,05 | 0,11 | 0,052 | 0,57 | 2,3 | 15,3 | 0 |
| Schnitzel vegetarian based on milk filled w cheese unprepared fortified w iron (3040) | 26 | 0 | 3,1 | 0,03 | 0,1 | 0,013 | 0,42 | 2,7 | 15 | 0 |
| Gouda cheese 48+ (513) | 333 | 0,3 | 0,9 | 0,01 | 0,28 | 0,036 | 2,03 | 5,1 | 24,9 | 0 |
| Sausage roast - vegetarian based on peas* (5563) | 9 | 0 | 4,6 | 0,08 | 0,07 | 0,051 | 0 | 2,8 | 21,1 | 0 |
| Vegetarian meatballs/burgers based on soy/wheat, enriched with iron and vitamin B12 (5515) | 51 | 0 | 2,8 | 0,05 | 0,06 | 0,07 | 0,62 | 0,8 | 22,4 | 0 |
| Egg salad (1499) | 54 | 1,4 | 17,1 | 0,04 | 0,17 | 0,092 | 1,23 | 2,7 | 47,4 | 0 |
| Peanut butter (455) | 0 | 0 | 12,4 | 0,13 | 0,1 | 0,02 | 0 | 9,6 | 81 | 0 |
| Spread sweet average (464) | 9 | 0 | 2,2 | 0,03 | 0,1 | 0,03 | 0,14 | 0,7 | 8 | 2 |
| Hummus (3207) | 1 | 0 | 3,5 | 0,07 | 0,04 | 0,107 | 0 | 1,8 | 30,2 | 2 |
| Sausage sandwich - vegetarian enriched with iron and vitamin B12 (2541) | 0 | 0 | 6,4 | 0,01 | 0,07 | 0,005 | 1,28 | 2,1 | 14,4 | 7 |
| Cheese salad (5074) | 118 | 0,1 | 4,7 | 0,03 | 0,1 | 0,015 | 0,73 | 1,7 | 6,7 | 0 |
| Cream cheese (3368) | 344 | 0,3 | na | 0,04 | 0,28 | 0,074 | 0,3 | na | 23 | 0 |
| Chicken egg (84) | 215 | 1,8 | 4,2 | 0,08 | 0,35 | 0,114 | 1,54 | 3,4 | 59,3 | 0 |
| Cheese 30+ (3164) | 199 | 0,2 | 0,5 | 0,03 | 0,33 | 0,05 | 2,23 | 6,7 | 35 | 0 |
| Cheese spread 45+ (2995) | 209 | 0,2 | 0,6 | 0,02 | 0,24 | 0,014 | 0,64 | 0,1 | 13 | 1 |
| Chocolate sprinkles (1963) | 4 | 0 | 0,8 | 0,02 | 0,05 | 0,018 | 0,21 | 1,6 | 10,2 | 0 |
| Cheese pastry (1488) | 56 | 0,2 | 0,8 | 0,05 | 0,16 | 0,04 | 0,51 | 1,6 | 11,4 | 0 |
| Baguette cheese-onion (2356) | 54 | 0,1 | 0,4 | 0,05 | 0,07 | 0,04 | 0,3 | 1,5 | 39,6 | 1 |
| Bread currant (233) | 0 | 0 | 0,7 | 0,06 | 0,11 | 0,12 | 0 | 1,3 | 29,1 | 0 |
| Roll white soft (230) | 1 | 0 | 0,2 | 0,08 | 0,1 | 0,041 | 0 | 1,4 | 43,3 | 0 |
| Lentils green/brown boiled (970) | 2 | 0 | 0,1 | 0,19 | 0,05 | 0,14 | 0 | 2,5 | 33 | 0 |
| Quorn minced meat (2030) | 0 | 0 | 0 | 0 | 0,45 | 0,059 | 0 | 4,2 | 45,4 | 0 |
| Vegetarian minced meat (2047) | 0 | 0 | 0,5 | 0,02 | 0,04 | 0,024 | 0 | 5,3 | 51,2 | 0 |
| Minced meat, vegetarian, based on soy, enriched with iron and vitamin B12. (5088) | 0 | 0 | 0,5 | 0,02 | 0,04 | 0,024 | 0,5 | 5,3 | 51,2 | 0 |
| Quorn pieces (2031) | 0 | 0 | 0 | 0,1 | 0,36 | 0,089 | 0 | 4,5 | 52,5 | 0 |
| Soup clear w vegetables and noodles (757) | 43 | 0 | 0,2 | 0,01 | 0,01 | 0,037 | 0 | 0,2 | 13,3 | 7 |
| Soup clear vegetables (759) | 44 | 0 | 0,2 | 0,01 | 0,01 | 0,036 | 0 | 0,2 | 13,3 | 7 |
| Soup thickened vegetables (763) | 61 | 0,2 | 0,8 | 0,01 | 0,01 | 0,036 | 0 | 0,2 | 13 | 6 |
| Soup thickened no filling (2561) | 29 | 0 | 0,1 | 0 | 0,01 | 0,003 | 0,02 | 0,1 | 0,5 | 0 |
| Crisp potato (122) | 7 | 0 | 4,3 | 0,21 | 0,08 | 0,72 | 0 | 4,5 | 12 | 11 |
| Cocktail snacks nibb-it (618) | 0 | 0 | na | 0 | 0,08 | na | 0 | 2,7 | 15 | 0 |
| Cassave crackers (2706) | 0 | 0 | 2,8 | 0,05 | 0,03 | 0,13 | 0 | 1,2 | 11 | 17 |
| Falafel (5574) | 14 | 0 | 1,7 | 0,15 | 0,05 | 0,16 | 0 | 2,2 | 35,6 | 0 |
| Tofu (5519) | 0 | 0,1 | 0,7 | 0,02 | 0,01 | 0,028 | 0 | 3,3 | 25,1 | 0 |
| Strips/pieces vegetarian based on soy/wheat enriched with iron and vitamin B12 (5554) | 2 | 0 | 4 | 0,07 | 0,11 | 0,03 | 0,6 | 5 | 39,9 | 0 |
| Vegan meat pieces* (5485) | 5 | 0 | 2,1 | 0,16 | 0,12 | 0,065 | 0 | 5 | 43,6 | 3 |
| Vegetarian schnitzel based on soy/wheat, enriched with iron and vitamin B12 (1512) | 8 | 0 | 2,3 | 0,08 | 0,08 | 0,121 | 0,54 | 4,5 | 48,1 | 3 |
| Sausage luncheon meat vegetarian * (5478) | 0 | 0 | 6,4 | 0,01 | 0,07 | 0,005 | 0 | 0,2 | 14,4 | 7 |
| Sesame paste tahin (1461) | 0 | 0 | 2,5 | 0,32 | 0,13 | 0,351 | 0 | 9 | 99 | 0 |
| Plant-based alternative to Gouda cheese based on coconut oil* (5465) | 52 | 0 | 0,3 | 0,01 | 0,05 | 0,005 | 0 | 0,1 | 0 | 10 |
| Bacon pieces vegetarian unprepared*  (5572) | 0 | 0 | 2,2 | 0,04 | 0,17 | 0,041 | 0 | 3,8 | 47,1 | 0 |
| Tea (645) | 0 | 0 | 0 | 0 | 0 | 0,018 | 0 | 0 | 3 | 0 |
| Coffee (644) | 0 | 0 | 0 | 0,01 | 0,01 | 0 | 0 | 0,4 | 0 | 0 |
| Drink soya original Alpro (870) | 0 | 0 | 0,4 | 0,02 | 0,02 | 0,052 | 0 | 0,9 | 13,9 | 0 |
| Water (1885) | 0 | 0 | 0 | 0 | 0 | 0 | 0 | 0 | 0 | 0 |
| Plant based alternative yoghurt enriched with vitamins (2888) | 0 | 0,8 | 0,4 | 0,01 | 0,01 | 0,037 | 0,35 | 1 | 21,5 | 0 |
| Drink oats – with calcium and vitamins (5427) | 0 | 1,1 | 0,4 | 0,02 | 0,21 | 0,005 | 0,36 | 0,1 | 2,5 | 0 |
| Drink soy-m sugar enriched with calcium and vitamins (1381) | 0 | 0,7 | 0,3 | 0,02 | 0,27 | 0,047 | 0,36 | 0,1 | 11,4 | 0 |
| Dessert soya Alpro (1380) | 4 | 0,8 | 0,4 | 0,02 | 0,21 | 0,034 | 0,38 | 0,7 | 9,4 | 0 |
| Ice cream coconut milk* (2939) | 0 | 0 | 0,2 | 0,01 | 0 | 0,003 | 0 | 0,4 | 0,7 | 0 |
| Cream based on veg oil Alpro cuisine (2262) | 0 | 0 | 4,6 | 0,02 | 0,01 | 0,036 | 0 | 0,7 | 9,7 | 0 |
| Cream based on veg oil Alpro Cuisine Light (3176) | 0 | 0 | 1,9 | 0,02 | 0,01 | 0,036 | 0 | 0,7 | 9,7 | 0 |
| Turkish delight (1364) | 0 | 0 | 0 | 0 | 0 | 0 | 0 | 0 | 0 | 0 |
| Popcorn sweet, popped wo oil (2387) | 4 | 0 | 0 | 0,3 | 0,09 | 0 | 0 | 1,7 | 0 | 0 |
| Chocolate dark w hazelnuts (2375) | 7 | 0 | 4,8 | 0,15 | 0,09 | 0,099 | 0,22 | 2,6 | 41,5 | 1 |
| Wafer galette (2425) | 197 | 1,9 | 5,7 | 0,05 | 0,03 | 0,03 | 0,12 | 1,1 | 10,5 | 0 |

| **Name and nevo code** | **Potassium (Mg)** | **Calcium (Mg)** | **Phosphorus (Mg)** | **Magnesium (Mg)** | **Iron (Mg)** | **Copper (Mg)** | **Selenium (Ug)** | **Zinc (Mg)** |
| --- | --- | --- | --- | --- | --- | --- | --- | --- |
| Chicken egg (83) | 145 | 57 | 229 | 14 | 2,3 | 0,06 | 17 | 1,56 |
| Mozzarella cheese  (1955) | 145 | 160 | 350 | 10 | 0,4 | NA | 3 | 2,6 |
| Vegetarian vegetable balls/burgers based on soy, enriched with iron and vitamin B12  (5551) | 570 | 66 | 150 | 46 | 4,1 | 0,18 | 8 | 0,82 |
| Vegetarian schnitzel, enriched with iron and vitamin B12 (5565) | 390 | 490 | 330 | 18 | 2,7 | 0,04 | 5 | 0,96 |
| Vegetarian vegetable balls/burgers based on soy* (5549) | 650 | 83 | 170 | 58 | 2,7 | 0,2 | 8 | 1,2 |
| Vegetarian burger with cheese (5566) | 320 | 140 | 110 | 20 | 0,6 | 0,07 | 6 | 0,79 |
| Schnitzel vegetarian based on milk filled w cheese unprepared fortified w iron (3040) | 390 | 647 | 330 | 18 | 2,1 | 0,04 | 5 | 0,96 |
| Gouda cheese 48+ (513) | 80 | 819 | 542 | 33 | 0,2 | 0,04 | 12 | 3,47 |
| Sausage roast - vegetarian based on peas* (5563) | 480 | 56 | 170 | 25 | 3,6 | 0,22 | 8 | 1,5 |
| Vegetarian meatballs/burgers based on soy/wheat, enriched with iron and vitamin B12 (5515) | 750 | 64 | 150 | 42 | 4,5 | 0,2 | 6 | 0,9 |
| Egg salad (1499) | 87 | 25 | 99 | 7 | 2 | 0,06 | 17 | 1,36 |
| Peanut butter (455) | 575 | 45 | 318 | 145 | 1,8 | 0,66 | 9 | 2,6 |
| Spread sweet average (464) | 335 | 58 | 105 | 48 | 3,4 | 0,35 | 2 | 0,82 |
| Hummus (3207) | 130 | 29 | 116 | 62 | 2 | 0,31 | 6 | 1,17 |
| Sausage sandwich - vegetarian enriched with iron and vitamin B12 (2541) | 400 | 48 | 85 | 12 | 4,3 | 0,08 | 3 | 0,45 |
| Cheese salad (5074) | 57 | 275 | 203 | 23 | 0,3 | 0,03 | 6 | 1,26 |
| Cream cheese (3368) | 100 | 200 | 150 | 10 | 0,3 | NA | NA | NA |
| Chicken egg (84) | 141 | 64 | 247 | 15 | 2,4 | 0,07 | 20 | 1,63 |
| Cheese 30+ (3164) | 107 | 1033 | 667 | 40 | 0,2 | 0,08 | 15 | 4,47 |
| Cheese spread 45+ (2995) | 40 | 450 | 781 | 21 | 0,1 | 0,03 | 11 | 2 |
| Chocolate sprinkles (1963) | 845 | 36 | 184 | 119 | 8,1 | 0,98 | 4 | 1,27 |
| Cheese pastry (1488) | 76 | 148 | 137 | 15 | 0,3 | 0,03 | 3 | 0,7 |
| Baguette cheese-onion (2356) | 128 | 145 | 149 | 20 | 0,6 | 0,09 | 4 | 0,95 |
| Bread currant (233) | 387 | 56 | 98 | 27 | 1,5 | 0,3 | 3 | 0,63 |
| Roll white soft (230) | 176 | 42 | 102 | 22 | 0,9 | 0,12 | 5 | 0,71 |
| Lentils green/brown boiled (970) | 657 | 23 | 160 | 34 | 2,9 | 0,42 | 7 | 1,4 |
| Quorn minced meat (2030) | 79 | 187 | 250 | 43 | 0,3 | 0,3 | 4 | 6,23 |
| Vegetarian minced meat (2047) | 850 | 140 | 270 | 110 | 2,7 | 0,27 | 12 | 0,96 |
| Minced meat, vegetarian, based on soy, enriched with iron and vitamin B12. (5088) | 850 | 140 | 270 | 110 | 7 | 0,27 | 12 | 0,96 |
| Quorn pieces (2031) | 100 | 138 | 210 | 32 | 0,3 | 0,28 | 2 | 5,37 |
| Soup clear w vegetables and noodles (757) | 68 | 12 | 13 | 5 | 0,2 | 0,04 | 0 | 0,12 |
| Soup clear vegetables (759) | 67 | 12 | 11 | 4 | 0,2 | 0,03 | 0 | 0,08 |
| Soup thickened vegetables (763) | 68 | 12 | 12 | 4 | 0,2 | 0,04 | 0 | 0,09 |
| Soup thickened no filling (2561) | 14 | 4 | 8 | 1 | 0,1 | 0,03 | 0 | 0,05 |
| Crisp potato (122) | 1269 | 27 | 150 | 60 | 2,3 | 0,22 | 1 | 1,3 |
| Cocktail snacks nibb-it (618) | 660 | 20 | 120 | 43 | 1,2 | 0,18 | 1 | 0,6 |
| Cassave crackers (2706) | 207 | 38 | 177 | 73 | 1,6 | 0,43 | 10 | 1,42 |
| Falafel (5547) | 460 | 69 | 160 | 59 | 2,3 | 0,29 | 5 | 1,3 |
| Tofu (5519) | 140 | 100 | 210 | 100 | 2,4 | 0,24 | 2 | 1,3 |
| Strips/pieces vegetarian based on soy/wheat enriched with iron and vitamin B12 (5554) | 620 | 56 | 180 | 40 | 4,6 | 0,26 | 10 | 1,1 |
| Vegan meat pieces* (5485) | 600 | 100 | 230 | 80 | 3,9 | 0,13 | 5 | 1,2 |
| Vegetarian schnitzel based on soy/wheat, enriched with iron and vitamin B12 (1512) | 650 | 83 | 170 | 58 | 3,9 | 0,2 | 8 | 1,2 |
| Sausage luncheon meat vegetarian * (5478) | 400 | 48 | 85 | 12 | 3,5 | 0,08 | 3 | 0,45 |
| Sesame paste tahin (1461) | 480 | 37 | 564 | 353 | 9 | 1,5 | 34 | 5,4 |
| Plant-based alternative to Gouda cheese based on coconut oil* (5465) | 9 | 7 | 15 | 3 | 0,3 | 0 | 0 | 0,02 |
| Bacon pieces vegetarian unprepared*  (5572) | 420 | 100 | 180 | 12 | 3,9 | 0,19 | 9 | 0,72 |
| Tea (645) | 8 | 5 | 2 | 1 | 0 | 0,01 | 0 | 0,01 |
| Coffee (644) | 78 | 4 | 0 | 6 | 0,2 | 0,01 | 0 | 0,01 |
| Drink soya original Alpro (870) | 150 | 17 | 44 | 18 | 0,5 | 0,13 | 1 | 0,35 |
| Water (1885) | 0 | 5 | 0 | 1 | 0 | 0,02 | 0 | 0 |
| Plant based alternative yoghurt enriched with vitamins (2888) | 150 | 140 | 97 | 19 | 0,5 | 0,12 | 0 | 0,32 |
| Drink oats – with calcium and vitamins (5427) | 30 | 119 | 12 | 3 | 0 | 0,02 | 0 | 0,04 |
| Drink soy-m sugar enriched with calcium and vitamins (1381) | 160 | 110 | 79 | 14 | 0,3 | 0,09 | 0 | 0,22 |
| Dessert soya Alpro (1380) | 120 | 120 | 81 | 15 | 0,4 | 0,09 | 0 | 0,23 |
| Ice cream coconut milk* (2939) | 116 | 6 | 34 | 22 | 0,5 | 0,17 | 0 | 0,31 |
| Cream based on veg oil Alpro cuisine (2262) | 105 | 13 | 31 | 13 | 0,4 | 0,09 | 1 | 0,25 |
| Cream based on veg oil Alpro Cuisine Light (3176) | 105 | 13 | 31 | 13 | 0,4 | 0,09 | 1 | 0,25 |
| Turkish delight (1364) | 5 | 4 | 3 | 2 | 0,2 | 0,05 | 0 | 0,04 |
| Popcorn sweet, popped wo oil (2387) | 183 | 8 | 214 | 66 | 0,9 | 0,32 | 1 | 0,08 |
| Chocolate dark w hazelnuts (2375) | 480 | 78 | 193 | 107 | 3,3 | 1,2 | 4 | 1,54 |
| Wafer galette (2425) | 70 | 12 | 54 | 10 | 0,6 | 0,05 | 3 | 0,34 |

For both tables:
Note: data obtained from https://nevo-online.rivm.nl
