# Supplementary material for: Eliminating Animal Products from the Diet Puts Dutch Older Adults at Risk of Micronutrient Deficiencies: A Simulation Study
Source: Curr Dev Nutr. 2026 Jul 25;10(8):109446. doi: 10.1016/j.cdnut.2026.109446 (PMC13487607; doi:10.1016/j.cdnut.2026.109446)
Supplement: Supplementary file 2 [file mmc2.docx]

**Title**: Appendix 2: R-script used for simulations

Appendix of the study “Eliminating animal products from the diet puts Dutch older adults at risk of micronutrient deficiencies: A simulation study”

Authors: Borkent, Grootswagers, Mendoza Valbuena, de van der Schueren.

PLEASE CONTACT CORRESPONDING AUTHOR BEFORE USING THIS SCRIPT: Jos.borkent@han.nl

Newer updates are available making it more flexible

library(readxl)

#open file 'final diets' as a dataframe, name it 'final_diets'

#open the file productlist2 as dataframe

#open the 3rd sheet of file 'nevo codes ingedeeld' as dataframe, name it foodgroupsnevo

final_diets <- read_excel ("DNFCS.xlsx")

productlist2 <- read_excel ("productlist2.xlsx")

nevo_codes <- read_excel ("Replacements.xlsx")

View(nevo_codes)

View(final_diets)

View(productlist2)

\# Compare the vcp nevo code in df final_diets to the nevo code in df productlist2. If there is a 0 in productlist2 for the pescetarian, vegetarian or vegan diet, this product is not allowed.

\# If there is a 1 for the nevocode, this product is allowed in the diet

\# For each diet, apply the 0 or 1 from the nevo code in df productlist2 to each product in df final_diets

\# In df final_diets, column pescetarian, place a 0 or 1 for each nevocode existing in df productlist2

for (i in 1:nrow(final_diets)) {

match_index <- match(final_diets$nevo_code_vcp[i], productlist2$`nevo code`)

if (!is.na(match_index)) {

final_diets$pescetarian[i] <- productlist2$pescetarian[match_index]

}

}

\# In df final_diets, column vegetarian, place a 0 or 1 for each nevocode existing in df productlist2

for (i in 1:nrow(final_diets)) {

match_index <- match(final_diets$nevo_code_vcp[i], productlist2$`nevo code`)

if (!is.na(match_index)) {

final_diets$vegetarian[i] <- productlist2$vegetarian[match_index]

}

}

\# In df final_diets, column vegan, place a 0 or 1 for each nevocode existing in df productlist2

for (i in 1:nrow(final_diets)) {

match_index <- match(final_diets$nevo_code_vcp[i], productlist2$`nevo code`)

if (!is.na(match_index)) {

final_diets$vegan[i] <- productlist2$`vegan`[match_index]

}

}

##########

#generate random numbers for each diet

#in df final_diets, column assigned_number_pesc, place a random number between 0 and 1, with 2 decimals

set.seed(7)

random_numbers <-runif(nrow(final_diets), min = 0, max = 1)

random_numbers <- round(random_numbers, 2)

final_diets$`assigned_number_pesc`<- random_numbers

#in df final_diets, column assigned.number.veg, place a random number between 0 and 1, with 2 decimals

set.seed(8)

random_numbers <-runif(nrow(final_diets), min = 0, max = 1)

random_numbers <- round(random_numbers, 2)

final_diets$assigned.number.veg<- random_numbers

#in df final_diets, column assigned.number.vegan, place a random number between 0 and 1, with 2 decimals

set.seed(9)

random_numbers <-runif(nrow(final_diets), min = 0, max = 1)

random_numbers <- round(random_numbers, 2)

final_diets$assigned.number.vegan<- random_numbers

#in df final_diets, column random_flex_80, place a random number between 0 and 1, with 2 decimals

set.seed(10)

random_numbers <-runif(nrow(final_diets), min = 0, max = 1)

random_numbers <- round(random_numbers, 2)

final_diets$random_flex_80<- random_numbers

#in df final_diets, column random_flex_40, place a random number between 0 and 1, with 2 decimals

set.seed(11)

random_numbers <-runif(nrow(final_diets), min = 0, max = 1)

random_numbers <- round(random_numbers, 2)

final_diets$random_flex_40<- random_numbers

\# For each random number assigned, place

#in df final_diets, column assigned_number_pescetarian, depending on the number in df final_diets, column assigned_number_pesc

for (i in 1:nrow(final_diets)) {

if (!is.na(final_diets$`assigned_number_pesc`[i])) {

if (final_diets$`assigned_number_pesc`[i] >= 0.00 & final_diets$`assigned_number_pesc`[i] <= 0.08) {final_diets$assigned_number_pescetarian[i] <- 1

} else if (final_diets$`assigned_number_pesc`[i] >= 0.08 & final_diets$`assigned_number_pesc`[i] <= 0.16) {final_diets$assigned_number_pescetarian[i] <- 2

} else if (final_diets$`assigned_number_pesc`[i] >= 0.16 & final_diets$`assigned_number_pesc`[i] <= 0.25) {final_diets$assigned_number_pescetarian[i] <- 3

} else if (final_diets$`assigned_number_pesc`[i] >= 0.25 & final_diets$`assigned_number_pesc`[i] <= 0.33) {final_diets$assigned_number_pescetarian[i] <- 4

} else if (final_diets$`assigned_number_pesc`[i] >= 0.33 & final_diets$`assigned_number_pesc`[i] <= 0.41) {final_diets$assigned_number_pescetarian[i] <- 5

} else if (final_diets$`assigned_number_pesc`[i] >= 0.41 & final_diets$`assigned_number_pesc`[i] <= 0.5) {final_diets$assigned_number_pescetarian[i] <- 6

} else if (final_diets$`assigned_number_pesc`[i] >= 0.50 & final_diets$`assigned_number_pesc`[i] <= 0.58) {final_diets$assigned_number_pescetarian[i] <- 7

} else if (final_diets$`assigned_number_pesc`[i] >= 0.58 & final_diets$`assigned_number_pesc`[i] <= 0.66) {final_diets$assigned_number_pescetarian[i] <- 8

} else if (final_diets$`assigned_number_pesc`[i] >= 0.66 & final_diets$`assigned_number_pesc`[i] <= 0.75) {final_diets$assigned_number_pescetarian[i] <- 9

} else if (final_diets$`assigned_number_pesc`[i] >= 0.75 & final_diets$`assigned_number_pesc`[i] <= 0.83) {final_diets$assigned_number_pescetarian[i] <- 10

} else if (final_diets$`assigned_number_pesc`[i] >= 0.83 & final_diets$`assigned_number_pesc`[i] <= 0.91) {final_diets$assigned_number_pescetarian[i] <- 11

} else if (final_diets$`assigned_number_pesc`[i] >= 0.91 & final_diets$`assigned_number_pesc`[i] <= 1.00) {final_diets$assigned_number_pescetarian[i] <- 12

}

}

}

#in df final_diets, column assigned_number_vegetarian, depending on the number in df final_diets, column assigned.number.veg

for (i in 1:nrow(final_diets)) {

if (!is.na(final_diets$assigned.number.veg[i])) {

if (final_diets$assigned.number.veg[i] >= 0.00 & final_diets$assigned.number.veg[i] <= 0.08) {final_diets$assigned_number_vegetarian[i] <- 1

} else if (final_diets$assigned.number.veg[i] >= 0.08 & final_diets$assigned.number.veg[i] <= 0.16) {final_diets$assigned_number_vegetarian[i] <- 2

} else if (final_diets$assigned.number.veg[i] >= 0.16 & final_diets$assigned.number.veg[i] <= 0.25) {final_diets$assigned_number_vegetarian[i] <- 3

} else if (final_diets$assigned.number.veg[i] >= 0.25 & final_diets$assigned.number.veg[i] <= 0.33) {final_diets$assigned_number_vegetarian[i] <- 4

} else if (final_diets$assigned.number.veg[i] >= 0.33 & final_diets$assigned.number.veg[i] <= 0.41) {final_diets$assigned_number_vegetarian[i] <- 5

} else if (final_diets$assigned.number.veg[i] >= 0.41 & final_diets$assigned.number.veg[i] <= 0.50) {final_diets$assigned_number_vegetarian[i] <- 6

} else if (final_diets$assigned.number.veg[i] >= 0.50 & final_diets$assigned.number.veg[i] <= 0.58) {final_diets$assigned_number_vegetarian[i] <- 7

} else if (final_diets$assigned.number.veg[i] >= 0.58 & final_diets$assigned.number.veg[i] <= 0.66) {final_diets$assigned_number_vegetarian[i] <- 8

} else if (final_diets$assigned.number.veg[i] >= 0.66 & final_diets$assigned.number.veg[i] <= 0.75) {final_diets$assigned_number_vegetarian[i] <- 9

} else if (final_diets$assigned.number.veg[i] >= 0.75 & final_diets$assigned.number.veg[i] <= 0.83) {final_diets$assigned_number_vegetarian[i] <- 10

} else if (final_diets$assigned.number.veg[i] >= 0.83 & final_diets$assigned.number.veg[i] <= 0.91) {final_diets$assigned_number_vegetarian[i] <- 11

} else if (final_diets$assigned.number.veg[i] >= 0.91 & final_diets$assigned.number.veg[i] <= 1.00) {final_diets$assigned_number_vegetarian[i] <- 12

}

}

}

#in df final_diets, column whole_number_vegan, depending on the number in df final_diets, column assigned.number.vegan

for (i in 1:nrow(final_diets)) {

if (!is.na(final_diets$assigned.number.vegan[i])) {

if (final_diets$assigned.number.vegan[i] >= 0.00 & final_diets$assigned.number.vegan[i] <= 0.08) {final_diets$whole_number_vegan[i] <- 1

} else if (final_diets$assigned.number.vegan[i] >= 0.08 & final_diets$assigned.number.vegan[i] <= 0.16) {final_diets$whole_number_vegan[i] <- 2

} else if (final_diets$assigned.number.vegan[i] >= 0.16 & final_diets$assigned.number.vegan[i] <= 0.25) {final_diets$whole_number_vegan[i] <- 3

} else if (final_diets$assigned.number.vegan[i] >= 0.25 & final_diets$assigned.number.vegan[i] <= 0.33) {final_diets$whole_number_vegan[i] <- 4

} else if (final_diets$assigned.number.vegan[i] >= 0.33 & final_diets$assigned.number.vegan[i] <= 0.41) {final_diets$whole_number_vegan[i] <- 5

} else if (final_diets$assigned.number.vegan[i] >= 0.41 & final_diets$assigned.number.vegan[i] <= 0.50) {final_diets$whole_number_vegan[i] <- 6

} else if (final_diets$assigned.number.vegan[i] >= 0.50 & final_diets$assigned.number.vegan[i] <= 0.58) {final_diets$whole_number_vegan[i] <- 7

} else if (final_diets$assigned.number.vegan[i] >= 0.58 & final_diets$assigned.number.vegan[i] <= 0.66) {final_diets$whole_number_vegan[i] <- 8

} else if (final_diets$assigned.number.vegan[i] >= 0.66 & final_diets$assigned.number.vegan[i] <= 0.75) {final_diets$whole_number_vegan[i] <- 9

} else if (final_diets$assigned.number.vegan[i] >= 0.75 & final_diets$assigned.number.vegan[i] <= 0.83) {final_diets$whole_number_vegan[i] <- 10

} else if (final_diets$assigned.number.vegan[i] >= 0.83 & final_diets$assigned.number.vegan[i] <= 0.91) {final_diets$whole_number_vegan[i] <- 11

} else if (final_diets$assigned.number.vegan[i] >= 0.91 & final_diets$assigned.number.vegan[i] <= 1.00) {final_diets$whole_number_vegan[i] <- 12

}

}

}

#flexitarian diets

\# For each random number assigned, place a number in the next column

#flex80

#in df final_diets, column number_flex_80, depending on the number in df final_diets, column random_flex_80

for (i in 1:nrow(final_diets)) {

if (!is.na(final_diets$random_flex_80[i])) {

if (final_diets$random_flex_80[i] >= 0.00 & final_diets$random_flex_80[i] <= 0.20) {final_diets$number_flex_80[i] <- 0

} else if (final_diets$random_flex_80[i] > 0.20 & final_diets$random_flex_80[i] <= 0.27) {final_diets$number_flex_80[i] <- 1

} else if (final_diets$random_flex_80[i] > 0.27 & final_diets$random_flex_80[i] <= 0.34) {final_diets$number_flex_80[i] <- 2

} else if (final_diets$random_flex_80[i] > 0.34 & final_diets$random_flex_80[i] <= 0.40) {final_diets$number_flex_80[i] <- 3

} else if (final_diets$random_flex_80[i] > 0.40 & final_diets$random_flex_80[i] <= 0.47) {final_diets$number_flex_80[i] <- 4

} else if (final_diets$random_flex_80[i] > 0.47 & final_diets$random_flex_80[i] <= 0.54) {final_diets$number_flex_80[i] <- 5

} else if (final_diets$random_flex_80[i] > 0.54 & final_diets$random_flex_80[i] <= 0.60) {final_diets$number_flex_80[i] <- 6

} else if (final_diets$random_flex_80[i] > 0.60 & final_diets$random_flex_80[i] <= 0.67) {final_diets$number_flex_80[i] <- 7

} else if (final_diets$random_flex_80[i] > 0.67 & final_diets$random_flex_80[i] <= 0.74) {final_diets$number_flex_80[i] <- 8

} else if (final_diets$random_flex_80[i] > 0.74 & final_diets$random_flex_80[i] <= 0.80) {final_diets$number_flex_80[i] <- 9

} else if (final_diets$random_flex_80[i] > 0.80 & final_diets$random_flex_80[i] <= 0.87) {final_diets$number_flex_80[i] <- 10

} else if (final_diets$random_flex_80[i] > 0.87 & final_diets$random_flex_80[i] <= 0.94) {final_diets$number_flex_80[i] <- 11

} else if (final_diets$random_flex_80[i] > 0.94 & final_diets$random_flex_80[i] <= 1.00) {final_diets$number_flex_80[i] <- 12

}

}

}

#flex40

#in df final_diets, column number_flex_40, depending on the number in df final_diets, column random_flex_40

for (i in 1:nrow(final_diets)) {

if (!is.na(final_diets$random_flex_80[i])) {

if (final_diets$random_flex_40[i] >= 0.00 & final_diets$random_flex_40[i] <= 0.60) {final_diets$number_flex_40[i] <- 0

} else if (final_diets$random_flex_40[i] > 0.60 & final_diets$random_flex_40[i] <= 0.63) {final_diets$number_flex_40[i] <- 1

} else if (final_diets$random_flex_40[i] > 0.63 & final_diets$random_flex_40[i] <= 0.66) {final_diets$number_flex_40[i] <- 2

} else if (final_diets$random_flex_40[i] > 0.66 & final_diets$random_flex_40[i] <= 0.70) {final_diets$number_flex_40[i] <- 3

} else if (final_diets$random_flex_40[i] > 0.70 & final_diets$random_flex_40[i] <= 0.73) {final_diets$number_flex_40[i] <- 4

} else if (final_diets$random_flex_40[i] > 0.73 & final_diets$random_flex_40[i] <= 0.76) {final_diets$number_flex_40[i] <- 5

} else if (final_diets$random_flex_40[i] > 0.76 & final_diets$random_flex_40[i] <= 0.80) {final_diets$number_flex_40[i] <- 6

} else if (final_diets$random_flex_40[i] > 0.80 & final_diets$random_flex_40[i] <= 0.73) {final_diets$number_flex_40[i] <- 7

} else if (final_diets$random_flex_40[i] > 0.83 & final_diets$random_flex_40[i] <= 0.86) {final_diets$number_flex_40[i] <- 8

} else if (final_diets$random_flex_40[i] > 0.86 & final_diets$random_flex_40[i] <= 0.90) {final_diets$number_flex_40[i] <- 9

} else if (final_diets$random_flex_40[i] > 0.90 & final_diets$random_flex_40[i] <= 0.93) {final_diets$number_flex_40[i] <- 10

} else if (final_diets$random_flex_40[i] > 0.93 & final_diets$random_flex_40[i] <= 0.96) {final_diets$number_flex_40[i] <- 11

} else if (final_diets$random_flex_40[i] > 0.96 & final_diets$random_flex_40[i] <= 1.00) {final_diets$number_flex_40[i] <- 12

}

}

}

#assign the food group number to each product that should be replaced

#compare the VCP nevo code in df final_diets to the nevo codes in df nevo_codes, place the corresponding food group number in df final_diets, column food_group_number

#(the column food group number is at the end, the 33rd column)

for (i in 1:nrow(final_diets)) {

if (!is.na(final_diets$nevo_code_vcp[i])) {

match_index <- which(nevo_codes$`nevo code` == final_diets$nevo_code_vcp[i])

if (length(match_index) > 0) {

final_diets$food_group_number[i] <- nevo_codes$`food group number`[match_index[1]]

}

}

}

#For each food product that is not allowed in the vegan diet (a 0 in column df final_diets column vegan), place the corresponding food group number in the column assigned.alternative.vegan

for (i in 1:nrow(final_diets)) {

if (is.na(final_diets$vegan[i])) {

next

}

if (final_diets$vegan[i] == 0) {

final_diets$assigned.alternative.vegan[i] <- final_diets$food_group_number[i]

}

}

#For each food product that is not allowed in the vegetarian diet (a 0 in column df final_diets column vegetarian), place the corresponding food group number in the column assigned.alternative.veg

for (i in 1:nrow(final_diets)) {

if (is.na(final_diets$vegetarian[i])) {

next

}

if (final_diets$vegetarian[i] == 0) {

final_diets$assigned.alternative.veg[i] <- final_diets$food_group_number[i]

}

}

#For each food product that is not allowed in the pescetarian diet (a 0 in column df final_diets column pescetarian), place the corresponding food group number in the column pescetarian_food_groups

for (i in 1:nrow(final_diets)) {

if (is.na(final_diets$pescetarian[i])) {

next

}

if (final_diets$pescetarian[i] == 0) {

final_diets$pescetarian_food_groups[i] <- final_diets$food_group_number[i]

}

}

##########

#vegan diet

#Check if there is a food group number in df final_diets column assigned.alternative.vegan

#find the corresponding food group number in df alternatives column food group number.

#check the random number in df final_diets column whole_number_vegan and find the corresponding number in df alternatives column assigned number

#place the name of the product with the food group number and the random number in df alternatives column vegan alternatives into df final_diets column alternative vegan

for (i in 1:nrow(final_diets)) {

if (!is.na(final_diets$assigned.alternative.vegan[i])) {

match_index <- which(alternatives$`food group number` == final_diets$assigned.alternative.vegan[i] &

alternatives$`assigned number` == final_diets$whole_number_vegan[i])

if (length(match_index) > 0) {

final_diets$alternative_vegan[i] <- alternatives$`vegan alternatives`[match_index[1]]

}

}

}

#pescetarian diet

#Check if there is a food group number in df final_diets column pescetarian_food_groups

#find the corresponding food group number in df alternatives column food group number.

#check the random number in df final_diets column assigned_number_pescetarian and find the corresponding number in df alternatives column assigned number

#place the name of the product with the food group number and the random number in df alternatives column vegetarian alternatives into df final_diets column pescetarian_alternatives

for (i in 1:nrow(final_diets)) {

if (!is.na(final_diets$pescetarian_food_groups[i])) {

match_index <- which(alternatives$`food group number` == final_diets$pescetarian_food_groups[i] &

alternatives$`assigned number` == final_diets$assigned_number_pescetarian[i])

if (length(match_index) > 0) {

final_diets$pescetarian_alternatives[i] <- alternatives$`vegetarian alternatives`[match_index[1]]

}

}

}

#vegetarian diet

#Check if there is a food group number in df final_diets column assigned.alternative.veg

#find the corresponding food group number in df alternatives column food group number.

#check the random number in df final_diets column assigned_number_vegetarian and find the corresponding number in df alternatives column assigned number

#place the name of the product with the food group number and the random number in df alternatives column vegetarian alternatives into df final_diets column assigned_alternative_vegetarian

for (i in 1:nrow(final_diets)) {

if (!is.na(final_diets$assigned.alternative.veg[i])) {

match_index <- which(alternatives$`food group number` == final_diets$assigned.alternative.veg[i] &

alternatives$`assigned number` == final_diets$assigned_number_vegetarian[i])

if (length(match_index) > 0) {

final_diets$assigned_alternative_vegetarian[i] <- alternatives$`vegetarian alternatives`[match_index[1]]

}

}

}

#flex80 diet

#Check if there is a food group number in df final_diets column assigned.alternative.veg

#find the corresponding food group number in df alternatives column food group number.

#check the random number in df final_diets column number_flex_80 and find the corresponding number in df alternatives column assigned number

#place the name of the product with the food group number and the random number in df alternatives column vegetarian alternatives into df final_diets column alternative_flex_80

for (i in 1:nrow(final_diets)) {

if (!is.na(final_diets$assigned.alternative.veg[i])) {

match_index <- which(alternatives$`food group number` == final_diets$assigned.alternative.veg[i] &

alternatives$`assigned number` == final_diets$number_flex_80[i])

if (length(match_index) > 0) {

final_diets$alternative_flex_80[i] <- alternatives$`vegetarian alternatives`[match_index[1]]

}

}

}

#flex40 diet

#Check if there is a food group number in df final_diets column assigned.alternative.veg

#find the corresponding food group number in df alternatives column food group number.

#check the random number in df final_diets column number_flex_40 and find the corresponding number in df alternatives column assigned number

#place the name of the product with the food group number and the random number in df alternatives column vegetarian alternatives into df final_diets column alternative_flex_40

for (i in 1:nrow(final_diets)) {

if (!is.na(final_diets$assigned.alternative.veg[i])) {

match_index <- which(alternatives$`food group number` == final_diets$assigned.alternative.veg[i] &

alternatives$`assigned number` == final_diets$number_flex_40[i])

if (length(match_index) > 0) {

final_diets$alternative_flex_40[i] <- alternatives$`vegetarian alternatives`[match_index[1]]

}

}

}

#assign the NEVO code

#if there is a word in df final_diets, column alternative_vegan, find this word in df alternatives column vegan alternatives,

#and place the corresponding nevo code in df alternatives column nevo codes...8 into df final_diets column nevo.code.vegan

for (i in 1:nrow(final_diets)) {

if (!is.na(final_diets$alternative_vegan[i])) {

match_index <- which(alternatives$`vegan alternatives` == final_diets$alternative_vegan[i])

if (length(match_index) > 0) {

final_diets$nevo.code.vegan[i] <- alternatives$`nevo codes...8`[match_index]

}

}

}

#if there is a word in df final_diets, column pescetarian_alternatives, find this word in df alternatives column vegetarian alternatives,

#and place the corresponding nevo code in df alternatives column nevo codes...6 into df final_diets column nevo.code.pesc

for (i in 1:nrow(final_diets)) {

if (!is.na(final_diets$pescetarian_alternatives[i])) {

match_index <- which(alternatives$`vegetarian alternatives` == final_diets$pescetarian_alternatives[i])

if (length(match_index) > 0) {

final_diets$nevo.code.pesc[i] <- alternatives$`nevo codes...6`[match_index]

}

}

}

#if there is a word in df final_diets, column assigned_alternative_vegatarian, find this word in df alternatives column vegetarian alternatives,

#and place the corresponding nevo code in df alternatives column nevo codes...6 into df final_diets column nevo_code_vegetarian

for (i in 1:nrow(final_diets)) {

if (!is.na(final_diets$assigned_alternative_vegetarian[i])) {

match_index <- which(alternatives$`vegetarian alternatives` == final_diets$assigned_alternative_vegetarian[i])

if (length(match_index) > 0) {

final_diets$nevo_code_vegetarian[i] <- alternatives$`nevo codes...6`[match_index]

}

}

}

#if there is a word in df final_diets, column alternative_flex_80, find this word in df alternatives column vegetarian alternatives,

#and place the corresponding nevo code in df alternatives column nevo codes...6 into df final_diets column flex80diet_nevo

for (i in 1:nrow(final_diets)) {

if (!is.na(final_diets$alternative_flex_80[i])) {

match_index <- which(alternatives$`vegetarian alternatives` == final_diets$alternative_flex_80[i])

if (length(match_index) > 0) {

final_diets$flex80diet_nevo[i] <- alternatives$`nevo codes...6`[match_index]

}

}

}

#if there is a word in df final_diets, column alternative_flex_40, find this word in df alternatives column vegetarian alternatives,

#and place the corresponding nevo code in df alternatives column nevo codes...6 into df final_diets column flex40diet_nevo

for (i in 1:nrow(final_diets)) {

if (!is.na(final_diets$alternative_flex_40[i])) {

match_index <- which(alternatives$`vegetarian alternatives` == final_diets$flex80_diet[i])

if (length(match_index) > 0) {

final_diets$flex40diet_nevo[i] <- alternatives$`nevo codes...6`[match_index]

}

}

}

########

#fill a column with the whole diet

#use the assigned alternative for each diet, and fill the rest with the original VCP products.

#if there is a word in df final diets column pescetarian_alternatives, place this word into df final_diets column pescetarian_diet.

#if there is no word in the column pescetarian_alternatives, place the word from df final_diets column prod_desc into column pescetarian_diet

for (i in 1:nrow(final_diets)) {

if (!is.na(final_diets$pescetarian_alternatives[i])) {

final_diets$pescetarian_diet[i] <- final_diets$pescetarian_alternatives[i]

} else {

final_diets$pescetarian_diet[i] <- final_diets$prod_desc[i]

}

}

#if there is a word in df final diets column assigned_alternative_vegetarian, place this word into df final_diets column vegetarian_diet.

#if there is no word in the column assigned_alternative_vegetarian, place the word from df final_diets column prod_desc into column vegetarian_diet

for (i in 1:nrow(final_diets)) {

if (!is.na(final_diets$assigned_alternative_vegetarian[i])) {

final_diets$vegetarian_diet[i] <- final_diets$assigned_alternative_vegetarian[i]

} else {

final_diets$vegetarian_diet[i] <- final_diets$prod_desc[i]

}

}

#if there is a word in df final diets column alternative_vegan, place this word into df final_diets column vegan_diet.

#if there is no word in the column alternative_vegan, place the word from df final_diets column prod_desc into column vegan_diet

for (i in 1:nrow(final_diets)) {

if (!is.na(final_diets$alternative_vegan[i])) {

final_diets$vegan_diet[i] <- final_diets$alternative_vegan[i]

} else {

final_diets$vegan_diet[i] <- final_diets$prod_desc[i]

}

}

#if there is a word in df final diets column alternative_flex_80, place this word into df final_diets column flex80_diet.

#if there is no word in the column alternative_flex_80, place the word from df final_diets column prod_desc into column flex80_diet

for (i in 1:nrow(final_diets)) {

if (!is.na(final_diets$alternative_flex_80[i])) {

final_diets$flex80_diet[i] <- final_diets$alternative_flex_80[i]

} else {

final_diets$flex80_diet[i] <- final_diets$prod_desc[i]

}

}

#if there is a word in df final diets column alternative_flex_40, place this word into df final_diets column flex40_diet.

#if there is no word in the column alternative_flex_40, place the word from df final_diets column prod_desc into column flex40_diet

for (i in 1:nrow(final_diets)) {

if (!is.na(final_diets$alternative_flex_40[i])) {

final_diets$flex40_diet[i] <- final_diets$alternative_flex_40[i]

} else {

final_diets$flex40_diet[i] <- final_diets$prod_desc[i]

}

}

##########

#Add the corresponding nevo codes for the flex80 and flex40 diet

#add column nevo_code_flex80 and nevo_code_flex40

final_diets$nevo_code_flex80 <- NA

final_diets$nevo_code_flex40 <- NA

\# if there is a word in alternative_flex_80, find this word in df alternatives column vegetarian alternatives

\# return the matching nevo code from df alternatives column nevo codes...6 to the df final_diets column nevo_code_flex80

for (i in 1:nrow(final_diets)) {

if (!is.na(final_diets$alternative_flex_80[i])) {

match_index <- which(alternatives$`vegetarian alternatives` == final_diets$alternative_flex_80[i])

if (length(match_index) > 0) {

final_diets$nevo_code_flex80[i] <- alternatives$`nevo codes...6`[match_index]

}

}

}

\# if there is a word in alternative_flex_40, find this word in df alternatives column vegetarian alternatives

\# return the matching nevo code from df alternatives column nevo codes...6 to the df final_diets column nevo_code_flex40

for (i in 1:nrow(final_diets)) {

if (!is.na(final_diets$alternative_flex_40[i])) {

match_index <- which(alternatives$`vegetarian alternatives` == final_diets$alternative_flex_40[i])

if (length(match_index) > 0) {

final_diets$nevo_code_flex40[i] <- alternatives$`nevo codes...6`[match_index]

}

}

}

#fill a column with all nevo-codes

#if there is a nevo code in df final_diets column nevo.code.pesc, place this code into df final_diets column pescetariandiet_

#if there is no code in column nevo.code.pesc, place the code of nevo_code_vcp into column pescetariandiet_

for (i in 1:nrow(final_diets)) {

if (!is.na(final_diets$nevo.code.pesc [i])) {

final_diets$pescetariandiet_[i] <- final_diets$nevo.code.pesc[i]

} else {

final_diets$pescetariandiet_[i] <- final_diets$nevo_code_vcp[i]

}

}

#if there is a nevo code in df final_diets column nevo_code_vegetarian, place this code into df final_diets column vegetariandiet_nevo

#if there is no code in column nevo_code_vegetarian, place the code of nevo_code_vcp into column vegetariandiet_nevo

for (i in 1:nrow(final_diets)) {

if (!is.na(final_diets$nevo_code_vegetarian [i])) {

final_diets$vegetariandiet_nevo[i] <- final_diets$nevo_code_vegetarian[i]

} else {

final_diets$vegetariandiet_nevo[i] <- final_diets$nevo_code_vcp[i]

}

}

#if there is a nevo code in df final_diets column nevo.code.vegan, place this code into df final_diets column vegandiet_nevo

#if there is no code in column nevo.code.vegan, place the code of nevo_code_vcp into column vegandiet_nevo

for (i in 1:nrow(final_diets)) {

if (!is.na(final_diets$nevo.code.vegan [i])) {

final_diets$vegandiet_nevo[i] <- final_diets$nevo.code.vegan[i]

} else {

final_diets$vegandiet_nevo[i] <- final_diets$nevo_code_vcp[i]

}

}

#if there is a nevo code in df final_diets column nevo_code_flex80, place this code into df final_diets column flex80diet_nevo

#if there is no code in column nevo_code_flex80, place the code of nevo_code_vcp into column flex80diet_nevo

for (i in 1:nrow(final_diets)) {

if (!is.na(final_diets$nevo_code_flex80 [i])) {

final_diets$flex80diet_nevo[i] <- final_diets$nevo_code_flex80[i]

} else {

final_diets$flex80diet_nevo[i] <- final_diets$nevo_code_vcp[i]

}

}

#if there is a nevo code in df final_diets column nevo_code_flex80, place this code into df final_diets column flex80diet_nevo

#if there is no code in column nevo_code_flex80, place the code of nevo_code_vcp into column flex80diet_nevo

for (i in 1:nrow(final_diets)) {

if (!is.na(final_diets$nevo_code_flex40 [i])) {

final_diets$flex40diet_nevo[i] <- final_diets$nevo_code_flex40[i]

} else {

final_diets$flex40diet_nevo[i] <- final_diets$nevo_code_vcp[i]

}

}

#safe the df final_diets as excelfile final_diets

library(writexl)

write.xlsx(final_diets, "final.xlsx")
